# Low dose pediatric chest computed tomography on a photon counting detector system – initial clinical experience

**DOI:** 10.1007/s00247-022-05584-4

**Published:** 2023-01-13

**Authors:** Ilias Tsiflikas, Greta Thater, Isabelle Ayx, Jakob Weiss, Juergen Schaefer, Thomas Stein, Stefan O. Schoenberg, Meike Weis

**Affiliations:** 1 Department of Diagnostic and Interventional Radiology, University Hospital, Eberhard Karls University Tuebingen, Tuebingen, Germany; 2 Department of Clinical Radiology and Nuclear Medicine, University Medical Center Mannheim, Medical Faculty Mannheim, Heidelberg University, Theodor-Kutzer-Ufer 1-3, 68167 Mannheim, Germany; 3 Department of Radiology, Freiburg University Hospital, Freiburg, Germany

**Keywords:** ALARA, Chest, Children, Computed tomography, Image gently, Low dose, Phantom, Photon counting, Radiation, Ultra-low dose

## Abstract

**Background:**

With the clinical release of a photon counting detector-based computed tomography (CT) system, the potential benefits of this new technology need to be evaluated clinically. Literature concerning this new generation of detector is sparse, especially in the field of pediatric radiology. Therefore, this study outlines our initial experience with ultra-low dose chest CT imaging on the new photon counting CT system.

**Materials and methods:**

A pediatric phantom (1-year old, CIRS ATOM phantom, model 704 [CIRS-computerized imaging reference system, Norfolk, VA]) was scanned at different dose levels and different image quality levels to define a protocol for clinical examinations. Next, 20 consecutive pediatric non-contrast ultra-low dose chest CT examinations were evaluated for radiation dose and diagnostic image quality using a 4-point Likert-scale—1 = excellent, 4 = bad image quality—by two radiologists in a consensus reading. This retrospective analysis was approved by the local research ethics committee.

**Results:**

Chest CT examinations performed at ultra-low radiation dose (effective dose 0.19 ± 0.07 mSv; size-specific dose estimate 0.45 ± 0.14 mGy) in pediatric patients ages (2.6 ± 1.8 years) show good to excellent image quality for lung structures (1.4 ± 0.4) and moderate image quality for soft tissue structures (2.8 ± 0.2).

**Conclusion:**

Pediatric ultra-low dose chest CT examinations are feasible with the new generation photon counting detector-based CT system. The benefits of this technology must be evaluated for pediatric patients from the outset.

## Introduction

Technical developments in photon counting detector-based computed tomography (CT) led to the first clinically approved photon counting detector-based CT system. In principle, photon counting technology has several advantages in comparison to energy integrating detectors: As the intermediate step of conversion into visible light is omitted, each photon leads directly to an electrical signal. As the photon detection threshold is set to an energy level above electronic noise, the latter is substantially decreased, which can be used for dose reduction. Additionally, the spatial resolution is increased due to the detector architecture. Rajendran et al. [1] reported a dose reduction of more than 60% in paranasal sinus imaging when using a photon counting detector-based CT system with additional tin-filtering in comparison to an energy integrating detector system. Furthermore, the detector allows differentiation of various energy levels of the photons, which offers new possibilities for material decomposition [2].

With the arrival on the market of photon counting detector-based CT systems, the theoretical advantages must be validated in clinical practice. Various applications outside of pediatric diagnostics have already been evaluated. For example, Zhou et al. [3] investigated the reduction of metal artifacts and improved dose efficiency of photon counting detector-based CT in comparison to a conventional energy integrating detector system [3]. Hagen et al. [4] demonstrated a dose reduction in obese patients with similar or even improved image quality with the use of a photon counting detector-based CT system.

Due to their smaller size and higher sensitivity to ionizing radiation, pediatric patients especially could benefit from the advantages of the new technology [5]. In the past few years, low-dose protocols for modern dual-source systems have been evaluated [6, 7], whereas data on pediatric photon counting detector-based CT examinations is sparse. Chen et al. [8] reported a pediatric phantom-based study using a non-clinically approved system that evaluated different tube voltage and tube current interrelationships. The present work demonstrates our initial experience in pediatric chest imaging on a photon counting detector-based CT system. We hope this will facilitate the introduction of the technology into clinical routine for pediatric patients. Besides patient measurements, our study demonstrates phantom measurements at different image quality levels – a parameter recently introduced to achieve similar image quality between different scanners – to demonstrate dose behavior at different image quality levels.

## Materials and methods

### Study design and patient selection

This study consists of two parts: (I) phantom scans (1-year old, CIRS ATOM phantom, model 704 [CIRS-computerized imaging reference system, Norfolk, VA]) and (II) a retrospective analysis of pediatric chest CT scans. Twenty consecutive non-enhanced pediatric chest CT scans performed between December 2021 and March 2022 were included in this study to evaluate first experiences with the photon counting detector-based CT system. Informed consent was available for each patient and the study was approved by the local research ethics committee.

### Computed tomography parameters

All CT examinations were performed on a first-generation whole-body dual-source photon-counting CT system (NAEOTOM Alpha; Siemens Healthcare GmbH, Forchheim, Germany) without sedation. Whenever necessary, due to patient motion, a dedicated pediatric body positioning aid was used to maintain the body in position with the arms above the head. A 100 kilovoltage peak (kVp) topogram with additional tin filtering (100 Sn) and a reference tube current of 25 milliampere-seconds (mAs) was used. Integrated automatic tube current modulation was used for the scan. A gantry rotation time of 0.25 s and a pitch factor of 2.4 were set. Image quality level was varied in steps of 5 until image quality level 50 and in steps of 10 between image quality levels 50 and 100 to model dose dependency on image quality level. The lowest possible image quality level was 1. Effective dose, volume CT dose index (CTDIvol) and size-specific dose estimate (SSDE) were recorded for each image quality level. A linear regression model was calculated to define the correlation between image quality level and dose parameters. For patient scans, the image quality level was set to 10. Only non-contrast-enhanced chest scans were included in the study.

Raw data were reconstructed with an iterative reconstruction technique (Quantum iterative reconstruction, QIR, Siemens Healthcare GmbH, Forcheim, Germany) with a strength level of 3 and a 512 × 512 pixel matrix size with a slice thickness of 1 mm. For lung tissue, a Bl60, and for soft tissue, a Br40 convolution kernel was applied.

### Radiation dose

Values for mean CTDI_vol_ referenced to a 32 cm phantom, dose length product (DLP), and SSDE were received from the scanner. Effective dose was extracted from a dose management software (teamplay Dose; Siemens Healthcare GmbH) which uses Monte Carlo simulations (VirtualDose CT; Virtual Phantoms Inc., Albany, NY) as the basis of computation. Effective dose was calculated according to the IRCP Publication 103 recommendations [9].

### Subjective image quality

Two radiologists (G.T. and M.W., with 4 years and 8 years of experience, respectively, in pediatric thoracic CT) assessed subjective image quality in a consensus reading on an off-line workstation (Aycan Osirix Pro, version 3.14.006; Aycan, Würzburg, Germany). The readers evaluated the soft tissue and lung structures. They started with standard window settings but were permitted to change window width and level manually to simulate standard diagnostic procedure.

All images were evaluated for diagnostic usability. Assessment was performed according to a variation of the method described by Niemann et al. [10] using a 4-point Likert scale to rate the analyzability of lung and soft tissue structures. Grading was performed for each structure depending on noise and delineation of anatomical structures as follows: 1-excellent, no noise; 2-good, mild noise; 3-moderate, moderate noise; 4-non-diagnostic, marked noise. The following structures were evaluated on lung images: the pulmonary fissures, the proximal (main to subsegmental) bronchi and adjacent pulmonary vessels, the peripheral (beyond subsegmental) bronchi with adjacent pulmonary vessels and the detectability of vascular structures in the outermost 10 mm of subpleural space. For soft tissue, the following structures were analyzed: the thymus, the trachea with adjacent lymph nodes at the level of the aortic arc and the detectability of thoracic wall muscles and parts of the upper abdomen included in the scan.

For analysis, the mean grading score with standard deviation was calculated separately for soft tissue and lung structure analyses.

### Statistical analysis

All statistical calculations were performed on commercially available statistics software (Prism, version 8.4.3; GraphPad Software, La Jolla, CA). Values are given as mean ± standard deviation.

## Results

### Phantom measurements

#### Variation in image quality levels

**Fig. 1:**
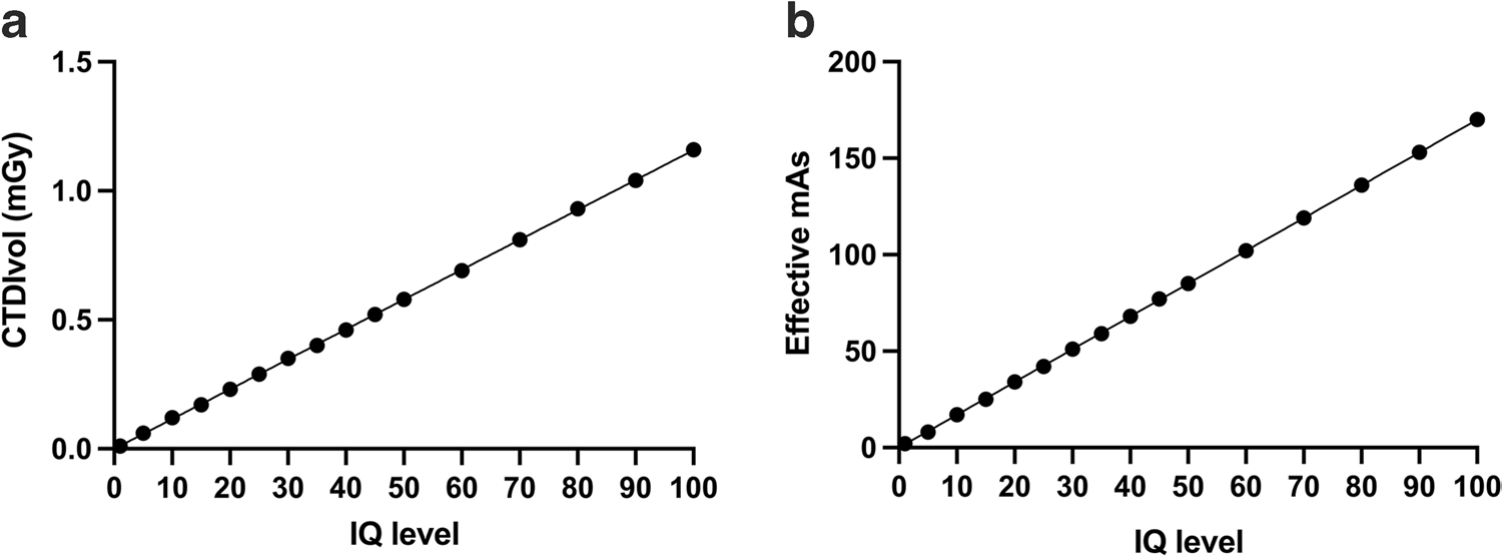
The linear relationship between image quality (IQ) level and volume computed tomography dose index (CTDI_vol_) (**a**) and effective mAs (**b**)

Increasing the new vendor-specific parameter of image quality – the image quality level (IQ level) – leads to a linear increase in effective mAs, accompanied by a linear increase in CTDI_vol_ (Fig. 1).

Linear correlation is highly significant (*P* < 0.001) and is described by the following equations (where IQ = image quality):

$$CTDIvol=0.01157\times IQ\;level$$

$$Effectivem\;As=1.7\times IQ\;level$$

Consequently, a two-fold increase in image quality level leads to a two-fold increase in dose.

#### Image quality

**Fig. 2:**
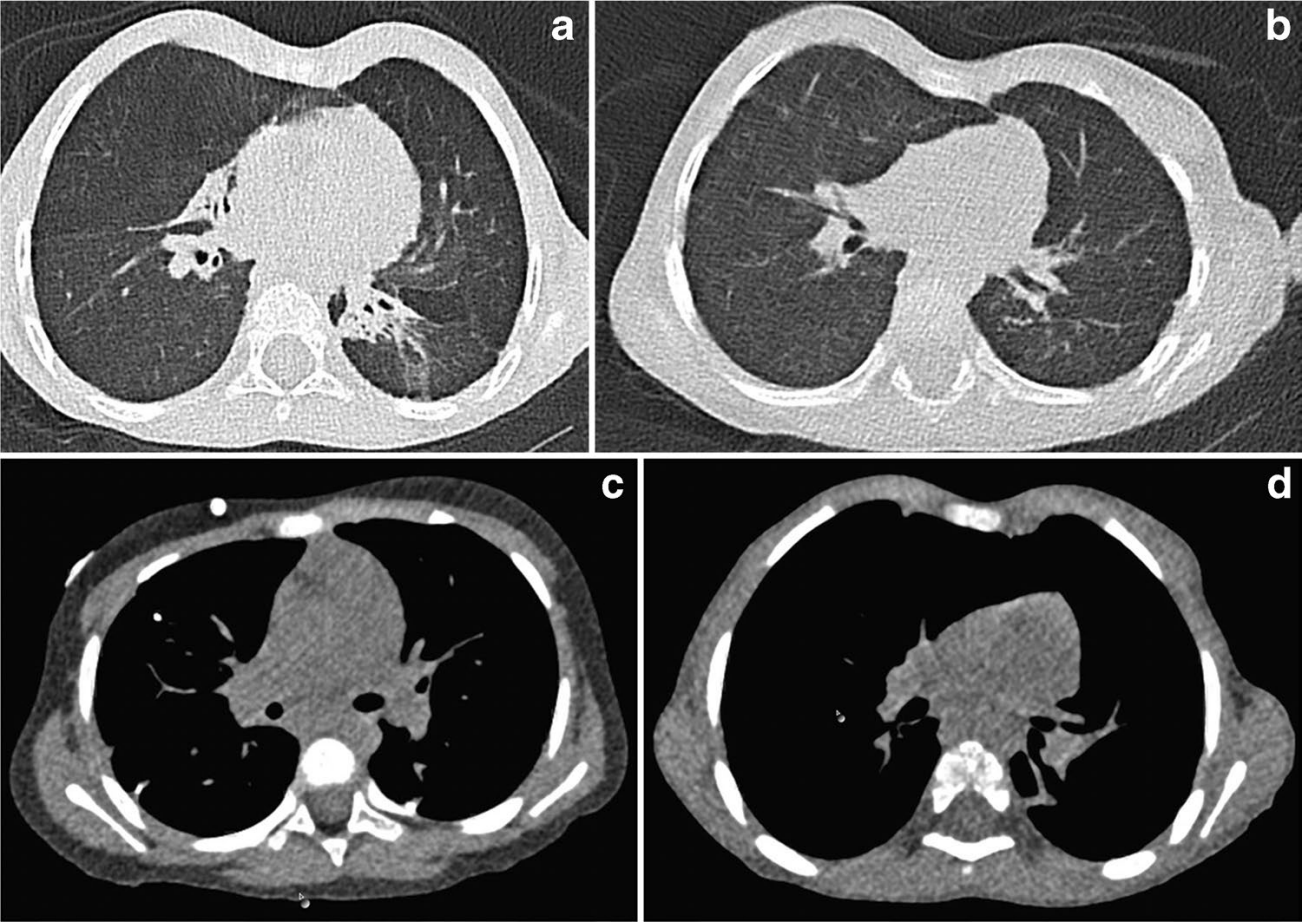
Spectrum of image quality scores. (**a**) Chest computed tomography scan reconstructed with lung kernel in a 2-year-old boy. Axial lung window image shows consolidations in the left lower and right middle lobe due to chronic aspiration caused by gastroesophageal reflux disease after congenital diaphragmatic hernia surgery. Lung structures are visible up to the periphery. Image quality rating for lung was 1. (**b**) Chest computed tomography scan reconstructed with lung kernel in a 4-year old boy following surgery for congenital diaphragmatic hernia. Axial lung window image shows blurred lung structures, especially in the periphery. Image quality rating for lung was 2.25. (**c**) Chest computed tomography scan reconstructed with soft tissue kernel in a 2-year old following resection of multiple neuroblastoma metastases. Axial mediastinal window image demonstrates that muscle delineation is possible, whereas mediastinal structures are not seen in detail. Image quality rating for soft tissue was 2.5. (**d**) Chest computed tomography scan reconstructed with soft tissue kernel in the same patient as in (**b**). Reconstructed axial mediastinal image shows restricted visualization of muscles and mediastinal structures because of blurring. Image quality rating for soft tissue was 3.25

All images were of diagnostic image quality (Fig. 2).

Image quality for lung tissue was rated as good to excellent (overall score for lung tissue 1.4 ± 0.4), while image quality for soft-tissue contrast was rated as moderate (2.8 ± 0.2; Table 1).

**Table 1:** Likert scores for various lung structures

	Mean Likert rating ± SD
Lung tissue contrast	
Fissures	1.53 ± 0.77
Proximal bronchi and adjacent pulmonary vessels	1.16 ± 0.37
Peripheral bronchi and adjacent pulmonary vessels	1.84 ± 0.76
Vascular structures within 10 mm of pleura	1.16 ± 0.37
Overall score for lung tissue	1.42 ± 0.44
Soft-tissue contrast	
Thymus	3.00 ± 0.65
Trachea and adjacent lymph node station	3.15 ± 0.37
Thoracic muscles	2.20 ± 0.52
Included upper abdomen	2.80 ± 0.41
Overall score for soft-tissue images	2.79 ± 0.23

Likert scores: 1-excellent, no noise; 2-good, mild noise; 3-moderate, moderate noise; 4-nondiagnostic, marked noise

*SD* standard deviation

#### Patient description

Of the 20 patients included in this study, 11 attended follow-up after congenital diaphragmatic hernia repair in the neonatal period, 6 attended follow-up after surgery for congenital pulmonary airway malformation, 2 were examined to evaluate pneumonia and 1 oncological patient was referred to CT for staging. The mean patient age was 2.6 ± 1.8 years. Radiation dose Table 2 describes patient dose parameters.

**Table 2:** Patient dose parameters

CTDI_vol_ (mGy)	0.21 ± 0.08
SSDE (mGy)	0.45 ± 0.14
DLP (mGyxcm)	4.93 ± 2.27
Effective mAs	32.20 ± 12.98
Effective dose (mSv)	0.19 ± 0.07

The figures after ± represent standard deviation

*CTDI*_*vo*l_ volume computed tomography dose index, *DLP* dose length product, *SSDE* size-specific dose estimate

## Discussion

Ultra-low dose thoracic chest CT can be performed with high image quality in pediatric patients on the new generation photon counting detector-based CT system.

As clinical experience with the new photon counting detector-based CT system is limited, especially in pediatric patients, we began with a phantom study to define the best chest CT protocol for our cohort. Given that the vendor had introduced a new unitless image quality parameter— the image quality level—we tested several quality levels and found a linear relationship between quality level and effective mAs and dose, respectively. Table 2 summarizes patient dose parameters.

By applying the dedicated chest CT protocol to pediatric chest CT scans, we were able to perform the examinations at very low doses (mean effective dose 0.19 ± 0.07 mSv, CTDI_vol_ 0.21 ± 0.08 mGy, SSDE 0.45 ± 0.14 mGy). Our dose values are in the same range as the “ultra-low dose” protocol (SSDE 0.4 ± 0.1 mGy) of Villanueva-Meyer et al. [11]. However, their ultra-low-dose protocol was only sufficient for some indications such as foreign body evaluation. In contrast, the image quality of our CT scans was good to excellent at identical dose values for all clinical questions concerning the lung parenchyma. This may be explained by the superior performance of the photon counting detector. Comparing dose values of the present work to values published by Esser et al. [6], those of the present work are lower. Additionally, dose values of this study are far below diagnostic reference levels in Germany, as shown by Schegerer et al. [12], in which a CTDI_vol_ of 1.7 mGy is given for a 1-year-old. Further, reported U.S. diagnostic reference values given for chest CT are much higher, with an achievable SSDE of 3.0 mGy for a thoracic CT without contrast enhancement [13].

It has been demonstrated that standardization of CT protocols in pediatric radiology departments leads to a significant decrease in radiation dose in comparison to non-pediatric radiology units [14]. For dose reduction, several CT parameters must be adjusted, as performed in this study: As demonstrated previously, additional tin filtering decreases dose significantly [15]. For example, Vivier et al. [16] demonstrated a reduction of image noise with the introduction of tin filtering at the same dose as low kV imaging at 70 kVp. Additionally, iterative reconstruction techniques should be applied in pediatric CT imaging, as used in this study. With the substantial decrease in image noise, the same image quality can be achieved at lower radiation dose [17]. Furthermore, high-pitch examinations decrease motion artifacts and reduce radiation dose significantly [6]. The techniques for dose reduction mentioned above are not available on all clinical CT systems, which leads to the difficulty of comparing dose levels between different scanner generations. Until the introduction of photon counting detector-based CT, modern dual-source scanners with energy integrating detectors were regarded as state-of-the-art technology; therefore, comparison between these technologies is worthwhile. A comparison of low kVp imaging and 100 kVp imaging with additional tin filtering on a modern dual-source system showed comparable low-dose values in pediatric patients (CTDI_vol_ 0.19 mGy [7]). To investigate the potential advantages of the new photon-counting detector for routine clinical practice, a direct comparison of objective and subjective image quality should be performed. Due to the excellent image quality of the lung tissue in our study, further dose reduction seems possible on the photon counting detector-based CT.

Additionally, spectral information is included in the photon counting detector-based CT signal and can be used for material decomposition and monoenergetic imaging—possibilities that were previously only available using dual-energy CT. Several applications, such as lung perfusion imaging and monoenergetic imaging for reduction of contrast agent dose, have already been successfully applied to pediatric patients [18]. In this context, photon counting technology seems promising for pediatric patients and should be further evaluated.

One limitation of our study is the sole inclusion of non-enhanced chest CT examinations, which was due to the low number of contrast-enhanced examinations and the absence of a low kVp option on the scanner during the study period, which would have been the preferred option for contrast-enhanced CT examinations [19]. The inclusion of non-enhanced CT scans explains the lower image quality for soft-tissue structures. Additionally, the low patient number could be criticized, however, this study was meant to be a feasibility study to guide pediatric radiologists in their first experiences with the new photon counting technology, for which purpose our small patient cohort seems adequate. Another limitation is that we did not calculate objective image quality parameters such as the signal-to-noise ratio (SNR). SNR is especially helpful when comparing different protocols and scanners, but this was beyond the scope of our study. Future work should focus on this.

## Conclusion

Ultra-low-dose chest CT examinations are feasible in pediatric patients using photon counting detector-based CT systems. As the technology seems promising in the context of dose-sparing and spectral information, its potential benefit should be further evaluated in pediatric patients.
